# Quality Assessment of Antenatal Care in Public Health Facilities of Nepal’s Hilly District: An Observational Study

**DOI:** 10.31729/jnma.v63i2091.9242

**Published:** 2025-11-30

**Authors:** Saraswoti Kumari Gautam Bhattarai, Wahid Abdullah Wajih, Sarala Pradhan Joshi, Basanta Sharma

**Affiliations:** 1 Institute of Medicine, Tribhuvan University, Maharajgunj, Kathmandu, Nepal; 2 Faculty of Medicine, University of Cyberjaya, Selangor, Malaysia; 3 Department of Obstetrics and Gynecology, Chitwan Medical College, Chitwan, Nepal

**Keywords:** *antenatal care*, *experience*, *pregnant women*, *quality of antenatal care*

## Abstract

**Introduction:**

Quality of antenatal care is essential for improving maternal and newborn health and plays a vital role in reducing maternal morbidity and mortality. This study aimed to assess perceptions of pregnant women regarding the quality of antenatal care.

**Methods:**

A facility-based descriptive study was conducted among 406 third trimester pregnant women using systematic random sampling. Semi-structured questionnaires were administered through face-to-face interviews in the Nepali language. Both low- and high-risk women who consented to participate were included. Ethical approval and informed consent were obtained. The data were analyzed using descriptive statistics.

**Results:**

Among the 406 pregnant women, 317 (78.07%) were between 20-35 years old, 255 (62.80%) were Brahman/Chhetri, and 380 (93.59%) followed Hinduism. About 240 (59.11%) belonged to Joint families, 369 (90.88%) were literate, 237 (58.37%) engaged in agriculture, and 181 (44.58%) were primigravida women. Overall, 287 (70.69%) reported receiving good quality antenatal care. Regarding the perception of quality care during pregnancy in different aspects, good quality care related to antenatal examination was received by 312 (76.84%), antenatal care by 339 (83.49%), 213 (52.46%) received adequate counselling and education, and 289 (71.18%) were satisfied with the service provision.

**Conclusions:**

Although the majority of pregnant women received good quality care during the antenatal period, gaps remained in counselling and education. Strengthening these components is essential to improve the overall quality of antenatal care services.

## INTRODUCTION

Antenatal care is vital for ensuring the health of mothers and newborns, as high-quality care leads to better outcomes.^1^ It connects women and their families to the health system, increasing the chance of having skilled care during childbirth and promoting long-term health. However, inadequate care breaks this continuity and puts both mothers and babies at risk.^2^

Although many women receive antenatal care, major gaps remain in communication between providers and women, and the availability of services.^3^ Improving antenatal care quality requires addressing the gaps and ensuring women’s experiences are heard and improved. While antenatal care prevents complications that threaten lives,^4^ Nepal’s maternal mortality rate is still high at 151 per 100,000 live births, exceeding the Sustainable Development Goals target.^5^

Therefore, this study aimed to assess pregnant women’s perception on the quality of antenatal care in the hilly district of Karnali province.

## METHODS

A facility-based descriptive study was conducted among pregnant women attending antenatal clinics at Birthing Centers, Primary Health Care Center, and Karnali Academy of Health Sciences in Jumla from April 2021 to July 2022. Ethical approval was obtained from the Nepal Health Research Council (Reg. no. 37/2021PhD).

A systematic random sampling technique was used to select the study participants from the total pregnant women attending antenatal care (ANC) services at the selected health facilities. A total of twelve health facilities were included in the study: Karnali Academy of Health Sciences Teaching Hospital, Kalika-khetu Primary Health Center (PHC), and ten birthing centers (Tatopani, Taliuma, Pandugupha, Hatsinja, Narakot, Raralihi, Depalgaun, Chhumchaur, Tamti, and Patarasi). Pregnant women in their third trimester, both low- and high-risk, who provided informed consent were included in the study, while those with chronic or systemic illnesses or mental health issues under treatment were excluded. The total sampling frame consisted of 1,759 pregnant women, according to ANC clinic records for the fiscal year 2019/2020. The sample size was calculated using the formula n = (Z^2^ × p × q) / e^2^, where Z = 1.96 at a 95% confidence interval, p = 0.38^6^ (prevalence), q = 1 - p = 0.62, and e = 0.05 (margin of error), resulting in 362. After adding a 10% non-response rate, the final sample size was 406. The sampling interval (K) was determined as N/n = 1759/406 = 4.3; therefore, every 4^th^ pregnant woman was selected from the ANC attendance register. The first participant was chosen randomly from among the first four using the lottery method, and subsequently, every 4^th^ case was included until the required sample size of 406 was achieved.

The research instrument was developed by the researcher, followed by a pretest to evaluate its reliability, and Cronbach’s alpha value was more than 0.75. After obtaining written informed consent, face-to-face interviews were conducted using a Nepali version questionnaire. Each interview lasted 20-25 minutes and included socio-demographic details and antenatal care-related questions. Data were entered into SPSS version 16 for analysis. Descriptive statistics was used for data analysis. For quality assessment, responses were scored as 1 for ‘yes’ and 0 for ‘no’; a total score of ≥75% indicated good quality antenatal care, while <75% indicated poor quality care.^7,8^

## RESULTS

There were 406 pregnant women included in the study, out of which, 317 (78.07%o) participants were 20 to 35 years age group, while 79 (19.45%) participants were less than 20 years age group. There were 255 (62.80%o) participants were from Brahman/Chhetri ethnicity, while 92 (22.66%) participants were from Dalit ethinicity. There were 240 (59.11%) participants who belonged to joint family and 380 (93.59%) participants belonged to Hindu religion. There were 151 (37.19%) participants who had read upto 9-12 class while 40 (9.85%) participants had read upto bachelors level. There were 181 (44.58%) participants who were primigravida while 202 (49.75%) were multigravida (Table 1).

Among 406 pregnant women, weight was measured in 390 (96.05%) participants, blood pressure in 393 (96.79%) participants, and anemia in eye and nail bed checked in 334 (82.26%) participants. For laboratory tests, urine protein was tested in 318 (78.32%) participants, blood haemoglobin and blood grouping in 355 (87.43%) participants, and HIV in 304 (74.87%) participants. Iron and folic acid were provided to 389 (95.81%) participants, Tetanus and Diphtheria (TD) immunization to 385 (94.82%) participants, and albendazole were provided to 385 (94.82%) participants. Regarding counselling, 396 (97.53%) women received advice on iron and folic acid, 340 (83.74%) women received advice on birth preparedness, and 367 (90.39%) on danger signs (Table 2).

**Table 1:** Sociodemographic characteristics of pregnant women (n = 406).

Sociodemographic Characteristics	n (%)
**Age (years)**	
< 20	79 (19.45)
20–35	317 (78.07)
> 35	10 (2.46)
**Ethnicity**	
Janjati	16 (3.94)
Dalit	92 (22.66)
Madhesi	1 (0.24)
Muslim	3 (0.73)
Brahman/Chhetri	255 (62.80)
Others	39 (9.60)
**Religion**	
Hindu	380 (93.59)
Muslim	5 (1.23)
Buddhist	9 (2.21)
Christian	12 (2.95)
**Type of family**	
Nuclear	162 (39.90)
Joint	240 (59.11)
Extended	4 (0.98)
**Educational attainment**	
Illiterate	37 (9.11)
Literate	66 (16.25)
1–8 class	99 (24.38)
9–12 class	151 (37.19)
Bachelor	40 (9.85)
Master and above	13 (3.20)
**Occupation**	
Agriculture	237 (58.37)
Homemaker	99 (24.38)
Business	28 (6.89)
Service	33 (8.12)
Labour work	2 (0.49)
Others	7 (1.72)
**Income sustainability**	
Up to 6 months	174 (42.85)
6–9 months	106 (26.10)
9–12 months	70 (17.24)
12 months and saving	56 (13.79)
**Gravida**	
Primigravida	181 (44.58)
Multigravida	202 (49.75)
Grand multigravida	23 (5.66)

About 268 (66.00%) of pregnant women waited no longer than 30 minutes to receive services, 360 (88.66%) participants reported discussions on the prevention and treatment of complications, 370 (91.13%) participants said their privacy was maintained during examinations and treatment, while 343(84.48%) participants said their confidentiality was maintained during examinations and 379 (93.34%) felt the providers treated them with respect. There were 358 (88.17%) participants who said there was availability of needed drugs in the health institutions during antenatal care (Table 3).

**Table 2:** Antenatal services received by pregnant women (n=406).

Antenatal service received	n(%)
Examinations*	
Weight checked	390(96.05)
BP checked	393(96.79)
Breast examined	290(71.42)
Hand and feet examined for edema	306(75.36)
Abdomen examined	387(95.32)
Eye and nailbed checked for anemia	334(82.26)
Antenatal care*	
Urine for protein checked	318(78.32)
Blood checked for Hb and Grouping	355(87.43)
Four-time ANC check-up	385(94.82)
Blood test for HIV	304(74.87)
Blood test for VDRL	277(68.22)
Received Iron and Folic Acid	389(95.81)
Received TD immunization	385(94.82)
Received the Albendazole drug	385(94.82)
Counselling and education*	
Counseling on the use of Iron and folic acid	396(97.53)
Counseling intake of the anthelminthic drug	390(96.05)
Counseling on TD immunization	384(94.58)
Counseling on preparation for childbirth	340(83.74)
Counseling on danger signs	367(90.39)
Counseling on breastfeeding	331(81.52)
Counseling on the care of a newborn baby	335(82.51)
Counseling on care in the postnatal period	338(83.25)
Counseling on family planning	323(79.55)

* Multiple Response

ANC = Antenatal Care; HIV = Human Immunodeficiency Virus; BP = Blood Pressure; Hb = Haemoglobin; TD = Tetanus and Diphtheria; VDRL = Venereal Disease Research Laboratory

**Table 3:** Services available in health institutions during antenatal care (n=406).

Services Available	n(%)
Waiting time <30 minutes to receive service	268(66.00%)
Discussed the complications of pregnancy	362(89.16)
Discussed on prevention and treatment of complications	360(88.66)
Privacy is maintained during examination and treatment	370(91.13)
Confidentiality is maintained during the examination	343(84.48)
Availability of needed drugs	358(88.17)
Service is available 7 days a week	102(25.12)
Service is available 24 hours and 7 days	99(24.38)
Cleanliness of a health institution	372(91.62)
Shows respectful behavior by a service provider	379(93.34)

**Table 4:** Experience of quality of antenatal care service among pregnant women (n=406).

Variables	Quality of antenatal care	
	Poor(%)	Good(%)
Antenatal examination	94(23.15)	312(76.84)
Antenatal care	67 (16.51)	339(83.49)
Antenatal counseling and education	193(47.54)	213(52.46)
Service provision at the antenatal clinic	117(28.82)	289(71.18)

Out of 406 pregnant women, 312 (76.84%) participants experienced good quality of antenatal examination in the health institutions. There were 289 (71.18%) women who experienced good service provided at the antenatal clinic provided by the health institutions while 193 (47.54%) poor service. However, 193 (47.54%) participants reported poor antenatal counseling and education provided by the service provided in the health institutions (Table 4).

Overall, out of 406 pregnant women, 287 (70.69%) reported receiving good quality antenatal care.

## DISCUSSION

In this study, among the 406 women, the majority, 287 (70.69%), reported good-quality antenatal care. Similarly previous study conducted in Nigeria reports 72.2% participants were satisfied with quality of antenatal care.^9^ This study found that 96.05% of women had their weight checked. In comparison, a study of Bangladesh reported that 88% of pregnant women underwent weight measurement.^10^ Similarly, blood pressure was checked in 96.79% of women in this study, which aligns with findings from India, where 97.1% underwent blood pressure monitoring.^11^ A study in Nepal also reported similar results, with 87.6% of women having their blood pressure measured and 92.2% having their weight checked.^12^ In this study, breast examinations were conducted in 71.42% of women, while hands and feet were assessed in 75.36%. Abdominal examinations were performed in 95.32% of cases, and 82.26% had their eyes and nailbeds examined for signs of anemia. Furthermore, urine tests for protein were conducted in 78.32%, and blood tests for hemoglobin and blood grouping were performed in 87.43% of women. A study carried out in a tertiary hospital, Kathmandu, showed that 90% of women received blood pressure monitoring, hemoglobin estimation, blood grouping, and Rhesus typing, as well as HIV and syphilis screening.^13^ Similar results were reported in an Indian study, where 93.5% underwent blood pressure monitoring, 94% had abdominal examinations, 93.5% had their weight checked, and 91.5% underwent laboratory investigations.^14^ In the present study, 94.82% did four antenatal check-ups, whereas a study conducted in the Sarlahi District of Nepal only 60% had four or more antenatal check-ups.^15^ One study finding of India revealed that 70.5% had four or more antenatal check-ups.^16^ These differences may be due to the socio-cultural background.

In the current study, blood tests were performed for HIV in 74.87% and for VDRL in 68.22% women. In contrast, a systematic review reported lower testing rates, with syphilis testing at 55.1% and HIV testing at 47.8%.^17^ Likewise, in the present study, 95.81% of women received iron and folic acid, 94.82% received tetanus-diphtheria immunization, and 94.82% received Albendazole. Similar findings were reported in a study from Kaski of Nepal, where 99.0% of pregnant women received iron/folic acid tablets and tetanus vaccination.^18^ However, another study from Nepal revealed lower coverage, with 66.6% of pregnant women received the tetanus vaccine, 66.6% received Albendazole, and only 54.16% received iron supplements during pregnancy,^19^ which is different from the current study.

In the present study, 94.82% received counselling on TD immunization, which is similar to a study done in Kaski, Nepal, which concluded that 99.0% received tetanus vaccination.^18^ Additionally, in this study, 83.74% of women received education and counseling on birth preparedness, whereas a study from India reported a much lower proportion, with only 24% received such services.^14^ Similarly, a study in Northwest Ethiopia found that only 14.4% of women were counseled on birth preparedness.^20^ Furthermore, in the current study, 90.39% of women were counseled about danger signs, which is similar to findings of a tertiary hospital in Kathmandu, Nepal, where 93% of pregnant women received counseling on danger signs.^13^ In contrast, a study from western Nepal reported that 74.4% of women were educated on danger signs during pregnancy, and 70.1% received education on danger signs during childbirth.^12^ Likewise, a study conducted in Bangladesh found that 71% of women received counseling on maternal and newborn danger signs.^21^ In the current study, 81.52% of women received counseling on breastfeeding, 82.51% on newborn care, and 83.25% on postnatal care. In contrast, a study from Sarlahi district, Nepal, showed that 70% of women did not receive breastfeeding counseling, and 67.5% did not receive counseling on postpartum care,^16^ which may reflect geographical and cultural differences between the districts. Additionally, 79.55% of women in this study received counseling on family planning, whereas a study from India, 63% received family planning education and counseling.^14^

In this study, 66.00% of pregnant women waited less than 30 minutes for services. In contrast, a study in Nigeria found that 78.2% waited up to 120 minutes for services.^4^ The Nepal Health Facility Survey reported that 54% of clients waited more than 30 minutes before receiving care.^22^ Additionally, in this study, 89.16% of women said that the service providers discussed pregnancy complications, and 88.66% reported discussions on prevention and treatment of complications. However, a study in Ethiopia found that only 60% received information on pregnancy-related complications.^23^ Another Ethiopian study showed that 74.2% of women felt health care providers communicated effectively with them.^7^

In this study, 91.13% of women reported that privacy was maintained during examination and treatment, contrasting with a study from a tertiary hospital in Kathmandu where only 47.1% reported privacy maintenance.^24^ However, a study from Ethiopia found a similar result, with 91.4% being satisfied with privacy.^25^ In this study, 91.62% found the facility clean, compared to 52.9% in a tertiary hospital of Nepal,^24^ while an Ethiopian study reported 87.5% satisfaction with cleanliness in maternity areas.^25^ Furthermore, 93.34% said providers showed respectful behavior, similar to a study from Sarlahi, Nepal, where 97.8% experienced respectful care.^15^ Finally, 95.32% received free services, while the study of a tertiary hospital in Nepal found 52.9% were satisfied with cleanliness and only 45.7% were satisfied with medicine availability.^24^

Among the 406 pregnant women in this study, 70.69% received overall good-quality antenatal care. This finding aligns with the study of Titilayo et al., where 69.4% had a positive perception of antenatal care quality.^9^ However, lower proportions were reported in studies by Bryce et al. and Tasneem & Ozdal, where only 49.1% and 48% of women, respectively, received good-quality care.^15, 26^ In terms of antenatal counseling and education, 52.46% in the current study experienced good quality, similar to Tasneem & Ozdal, where 46% reported adequate information sharing.^26^ In contrast, Hailu et al. found that only 33% received good-quality antenatal care.^7^

This study was limited to health facility settings; therefore, the findings of this study may not be generalizable to all pregnant women residing in the hilly district. Hence, future researchers are recommended to conduct community-based studies to obtain more comprehensive and representative results.

## CONCLUSION

Based on the findings of the study, the majority of the pregnant women reported as a perception of good quality of antenatal care. Although perceived overall care quality was good, the counselling and education aspect was lacking. Therefore, it is recommended that health facilities strengthen antenatal counselling and education to improve overall care quality.

## Data Availability

The data are available from the corresponding author upon reasonable request.
